# Potential associations of selected polymorphic genetic variants with COVID-19 disease susceptibility and severity

**DOI:** 10.1371/journal.pone.0316396

**Published:** 2025-01-03

**Authors:** Orsolya Mózner, Edit Szabó, Anna Kulin, György Várady, Judit Moldvay, Vivien Vass, Andrea Szentesi, Ágoston Jánosi, Péter Hegyi, Balázs Sarkadi

**Affiliations:** 1 Institute of Molecular Life Sciences, HUN-REN Research Centre for Natural Sciences, Budapest, Hungary; 2 Doctoral School, Semmelweis University, Budapest, Hungary; 3 1^st^ Department of Pulmonology, National Korányi Institute of Pulmonology; 4 Department of Pulmonology, University of Szeged Albert Szent-Györgyi Medical School; 5 Institute for Translational Medicine, University of Pécs, Medical School, Pécs, Hungary; 6 Institute of Pancreatic Diseases and Centre for Translational Medicine, Semmelweis University, Budapest, Hungary; 7 Heim Pál National Pediatric Institute, Budapest, Hungary; Banaras Hindu University, INDIA

## Abstract

In this study, we analyzed the potential associations of selected laboratory and anamnestic parameters, as well as 12 genetic polymorphisms (SNPs), with clinical COVID-19 occurrence and severity in 869 hospitalized patients. The SNPs analyzed by qPCR were selected based on population-wide genetic (GWAS) data previously indicating association with the severity of COVID-19, and additional SNPs that have been shown to be important in cellular processes were also examined. We confirmed the associations of COVID-19 with pre-existing diabetes and found an unexpected association between less severe disease and the loss of smell and taste. Regarding the genetic polymorphisms, a higher allele frequency of the *LZTFL1* and *IFNAR2* minor variants significantly correlated with greater COVID-19 disease susceptibility (hospitalization) and severity, and a similar tendency was observed for the *RAVER1* and the *MUC5B* variants. Interestingly, the *ATP2B4* minor haplotype, protecting against malaria, correlated with an increased disease susceptibility, while in diabetic patients disease susceptibility was lower in the presence of a reduced-function ABCG2 transporter variant. Our current results, which should be reinforced by larger studies, indicate that together with laboratory and anamnestic parameters, genetic polymorphisms may have predictive value for the clinical occurrence and severity of COVID-19.

## Introduction

The course of the COVID-19 pandemic has shown great individual variability, from asymptomatic infection or mild disease to severe disease, in some cases leading to patient death. In addition to direct viral toxicity, hyperreactivity of the immune system may significantly worsen patients’ conditions, and endothelial damage, microvascular injury, and hypercoagulability with thrombosis may also occur. In addition to the signs of severe pulmonary disease, the poor prognosis of hospitalized patients is predominantly indicated by laboratory data reflecting an overreaction of the immune system to viral infection. The already established factors affecting the severity of acute COVID-19 include older age, overweight status, and comorbidities (e.g., diabetes) [1–4]. The currently used pharmacological agents to treat COVID-19 have questionable effects in severe cases, and positive results are mostly observed at the initial stage of this disease (see [5–9]).

Increasing amounts of data are available about the potential effects of individual genetic backgrounds on the course and severity of COVID-19. Genetic factors affecting disease severity include molecular switches of the cellular immune response, e.g., the double-stranded RNA sensor Toll-like receptor 3 (TLR3) and type I interferon (IFN)-related pathways (see [10–14]). Rare mutations, such as loss-of-function deletions in the genes coding for the participants of these pathways, causing deficiencies in the expression of TLR3, TICAM1 (Toll Like Receptor Adaptor Molecule 1), IRF3 and IRF7 (Interferon Regulatory Factors 3 and 7), and IFNAR1 and IFNAR2 (Interferon Alpha and Beta Receptor subunits 1 and 2), have been described as genetic factors underlying severe pneumonia in patients with COVID-19 [11, 12].

At the population level, rare mutations may not affect the clinical development of acute or chronic COVID-19. In contrast, the presence of certain single nucleotide polymorphisms (SNPs) and related complex haplotypes (a series of coinherited SNPs) is closely associated with the clinical course of COVID-19 in GWA (genome-wide association) studies (see [14]), especially regarding severe lung inflammation and the consequently required ICU (intensive care unit) treatment. Interestingly, several such haplotypes originate from the genetic material of the Neanderthal man, as segments (haplotypes) of the Neanderthal genome with characteristic SNPs are still present in the human genome. The Neanderthal haplotypes are almost absent in African populations (at the origin of modern humans) but present with variable frequencies in various other geographical regions (see [15, 16]).

One of these Neanderthal haplotypes is located on chromosome 3, with a large number of coinherited (haplotype) SNPs (chr3p21.31 –lead SNP: rs73064425), and the region includes six genes *(SLC6A20*, *LZTFL1*, *CCR9*, *FYCO1*, *CXCR6* and *XCR1)*, which are potentially important in the immune response to this viral disease. The *CCR9*, *CCR6* and *XCR1* genes encode cytokine receptors, which may be involved in the “cytokine storm” in COVID-19 patients. The LZTFL1 protein (encoding chr3, p21.31 –lead SNP: rs73064425) regulates ciliary transport processes in the airways and is potentially an important factor in the treatment of COVID-19 [17–24]. As reported in GWAS, the presence of this Neanderthal-related haplotype correlates with doubling of the occurrence of severe respiratory disease in COVID-19 patients [15, 16].

Another Neanderthal-originating gene fragment, suspected to correlate with the severity of COVID-19, is a haplotype within the dipeptidyl peptidase 4 *(DPP4-DT)* gene on chromosome 2 (q24.2) with the leading SNP rs117888248/rs118098838. The DPP4 protein, together with ACE2, was found to be one of the binding sites of the SARS-CoV-2 virus, and the presence of the *DPP4-DT* Neanderthal-related haplotype in a heterozygous form was reported to double the appearance of severe COVID-19, while in a homozygous form, it correlated with a quadruple occurrence of severe disease [15, 16, 25, 26].

Other Neanderthal-related haplotypes reported to correlate with the severity of COVID-19 include genetic elements coding for the DPP9 protein (chr19, p13.3 –lead SNP: rs2109069) and the interferon alpha receptor (IFNAR2) protein (coding chr21, q22.1 –lead SNP: rs2236757) (see refs [27, 28]). In OAS1, the OAS2 and OAS3 protein coding regions of a haplotype (chr12, q24.13; lead SNP: rs10735079) were reported to correlate with less severe disease, while the protection against severe disease conferred by the Neanderthal *OAS* locus was substantially lower than the increased risk conferred by the chromosome 3 locus (refs [15, 16]). These interferon-induced OAS proteins produce short polyadenylates from RNA based on their ribonuclease activity and have antiviral effects in virus-infected cells.

In addition to the genes related to this Neanderthal heritage site, some relatively frequent SNPs in membrane receptor or transporter proteins may also have significant effects on acute or long-term COVID-19. The ACE2 membrane protein is the key receptor for SARS-CoV-2 binding to its cellular targets, and a haplotype in the *ACE2* gene (chrX, p22.2 –lead SNP rs2285666) has been shown to reduce the expression of this receptor. The variable presence of the T/A and G/C alleles may contribute to malaria sensitivity [29] and has been implicated in affecting COVID-19 susceptibility [30]. Similar to *ACE2*, *RAVER1* (chr191, p13.2– lead SNP: rs74956615) is a risk factor for COVID-19 infection [17, 18, 31]. Mucin encoded by *MUC5B* (chr11, p15.5 –lead SNP: rs35705950) is an important component of the innate immune response, and the presence of its promoter polymorphism, rs35705950, has a positive effect on the outcome of lung diseases through increased expression of MUC5B [32–34]. Loss of sense of smell (anosmia) or taste (ageusia) are characteristic symptoms of COVID-19 and are the earliest and most frequently reported indicators of the acute phase of SARS-CoV-2 infection. These symptoms have been reported to be variably associated with recovery, and the polymorphism of *UGT2A1* (chr4, q13.3– lead SNP: rs7688383) is one of the most significant genetic markers associated with loss of smell and taste [35, 36].

In addition to SNPs that have been previously studied in the context of COVID-19, our research also focused on genetic variations in membrane transporter proteins known to play key roles in cellular processes. Genetic polymorphisms in the PMCA4b protein, which widely affect cellular calcium homeostasis (a minor haplotype (referred to as “*ATP2B4*.haplo1”) in the regulatory region of the corresponding *ATP2B4* gene, chr1, q32.1—lead SNP: rs1541252) (see [37]), or SNPs within GLUT1, a key cellular transporter protein of glucose and vitamin C (encoded by the *SLC2A1* gene, chr1, p34.2—lead SNP: rs1385129 [38]), may modulate disease severity by affecting general metabolism. A frequent polymorphic genetic variant of the ABCG2 transporter (chr4, q22.1 –SNP rs2231142, resulting in a Q141K amino acid change) reduces uric acid, xenobiotic, and drug transport in various tissues and tissue barriers [39, 40].

While genome-wide association (GWA) studies are useful for exploring the role of potential genetic factors in large populations, small effects increasing disease risk in many cases cannot be distinguished from background noise, and only genetic variants with strong statistical evidence can be considered significant. Therefore, targeted molecular genetic studies should help to establish firm and relevant connections between selected polymorphic variations and the course of COVID-19, which affects numerous tissues and organs.

In this work, based on existing (although at that time mostly preliminary) data, we selected and analyzed 12 haplotypes and lead SNPs (see S1 Table in S1 File) potentially relevant to the reaction of the human body to this viral disease in 869 hospitalized patients in Hungary. At the time of data collection (between 2020 and 2021), the dominant SARS-CoV-2 variants were the original Wuhan variant and the Delta variant, and there was no effective vaccination or treatment for this disease.

Although only a limited number of potentially relevant genetic variants were evaluated in a relatively small number of patients, the present study may help to decipher the role of several clinical, anamnestic and genetic parameters in the occurrence and severity of this viral disease. Additionally, when extended to a larger number of patients, our results may provide a personalized tool for assessing the expected course, severity, and long-term, chronic effects in COVID-19 patients.

## Methods

### Clinical samples

Detailed clinical data were entered into this database by the participating clinicians at the Korányi Clinic, led by Judit Moldvay, and the University of Pécs, with the leadership of Péter Hegyi. The patients were informed about the research project, and written consent was obtained to participate in this study. All methods were performed in accordance with the relevant guidelines and regulations. Ethical permission from ETT TUKEB, NNK 24004-7/2021/EihO, and 20800-6/2020/EÜIG was obtained to perform these noninvasive molecular genetic studies. Clinical data and blood samples were collected between 2020 and 2021; at that time, no vaccination was accessible, and the collection did not include effectively vaccinated patients. The patients’ disease conditions (mild, moderate, severe or critical) were assessed by the clinical team based on clinical and laboratory parameters according to the WHO classification (Clinical management of COVID-19: Living guidance—No. WHO/2019-nCoV/clinical/2021.2). As addressed in some cases during the analysis, this grouping was further simplified into a two-group system, and the severe+critical groups were compared to the mild+moderate groups.

The anonymized data presented in this study, as well as those of the genetic analyses, are available in the data repository upon request.

### Genetic analysis

In this work, we studied the prevalence of specific human SNPs and their potential alteration frequency in (n = 869) COVID-19 patients. The SNPs studied here are summarized in S1 Table in S1 File.

We prepared genomic DNA from blood samples obtained from COVID-19 patients with variable clinical courses (see above). Blood samples (1 mL) were collected in EDTA tubes during routine laboratory testing (without any additional burden to the patients). Genomic DNA was purified from 300 μL of EDTA-anticoagulated blood samples with a Puregene Blood Kit (Qiagen). TaqMan-based qPCRs for SNPs (for details, see S2 Table in S1 File) were performed in a StepOnePlus device (Applied Biosystems) with premade assay mixes and a master mix (cat. 4371353) from Thermo Fisher. The results of the molecular genetic studies and the clinical and laboratory data were collected in an anonymized database and subjected to detailed statistical analysis. Samples with incomplete data were excluded from the final analysis. This was due to either the failure of the TaqMan PCR or the lack of clear documentation regarding specific symptoms (e.g., due to the patient’s poor condition).

In two cases the SNPs examined in the patients (*IFNAR2* rs2236757 and *LZTFL1* rs73064425*)* have also been analyzed in the pre-COVID-19 DNA samples of healthy Hungarian volunteers (282 samples), collected in the work reported earlier [39].

### Statistical analysis

The European minor allele frequencies (1000 Genome (phase3 release V3+) and the ALFA (Release Version: 20230706150541) databases) were collected from the NCBI dbSNP database (downloading date: 10/1/2024, https://www.ncbi.nlm.nih.gov/snp/). Statistical analysis was conducted with GraphPad Prism 8.0.1.

The odds ratios (ORs) and 95% confidence intervals (CIs) for the associations between severity and the SNPs and/or comorbidities were calculated by logistic regression (R Studio, version number: 4.1.3). The comparisons of the allele frequencies with the European population values were analyzed by using Fisher’s exact test (p<0.05), and the ORs with confidence intervals were analyzed by the Baptista-Pike test (Prism 8.0.1, GraphPad). The number of patients (n) involved in each analysis is indicated in the respective tables. When comparing observed allele frequencies with European reference data from the 1000 Genome and ALFA databases, Bonferroni correction for multiple comparisons was performed. Adjusted p-values (p-value × number of SNPs examined (12)) are indicated in the S1 File; p-values that remained <0.05 after Bonferroni adjustment are marked with a * in Table 1.

**Table 1:** Allele frequencies of minor variants (MAFs) in the general population and hospitalized COVID-19 patients. Odds ratios and significance. Allele frequencies of the hospitalized COVID-patients determined in this study were compared to the 1000 Genome (phase3 release V3+) and the ALFA (Release Version: 20230706150541) European allele frequencies. P- values marked with a * are still significant after Bonferroni adjustment for multiple comparisons.

*Gene polymorphism*	*Minor variant -reported COVID association*	*MAF (EUR)*	*MAF COVID hospitalized*	*Ref*.: *1000Genomes (EUR)*		*Ref*.: *ALFA (EUR)*	
	* *	*(ALFA/1000G)*	* *	*p value*	*OR (95% CI)*	*p value*	*OR (95% CI)*
***DPP4_DT*** ***rs118098838***	***More severe disease*** *[14]*	***0*.*0050/0*.*0099***	***0*.*0076***	***0*.*6172***	***0*.*70 (0*.*26–1*.*99)***	***0*.*4504***	***1*.*41 (0*.*66–3*.*09)***
***DPP9*** ***rs2109069***	***More severe disease [14]***	***0*.*3088/0*.*3211***	***0*.*2963***	***0*.*2492***	***0*.*89 (0*.*73–1*.*08)***	***0*.*4388***	***0*.*94 (0*.*81–1*.*09)***
***OAS3*** ***rs10735079***	***More severe disease*** *[14]*	***0*.*3662/0*.*3638***	***0*.*3267***	***0*.*0967***	***0*.*85 (0*.*70–1*.*03)***	***0*.*0171***	***0*.*84 (0*.*73–0*.*97)***
***IFNAR2*** ***rs2236757***	***More severe disease*** *[28]*	***0*.*2941/0*.*2942***	***0*.*3575***	*0*.0040*	***1*.*33 (1*.*10–1*.*62)***	*<0*.0001*	***1*.*33 (1*.*16–1*.*53)***
***LZTFL1*** ***rs73064425***	***More severe disease*** *[18]*	***0*.*0820/0*.*0795***	***0*.*1545***	*<0*.0001*	***2*.*11 (0*.*79–2*.*82)***	*<0*.0001*	***2*.*04 (1*.*68–4*.*03)***
***ACE2*** ***rs2285666***	***Lower infection rate [30]***	***0*.*2038/0*.*2350***	***0*.*2022***	***0*.*1171***	***0*.*82 (0*.*65–1*.*05)***	***0*.*9321***	***0*.*99 (0*.*84–1*.*17)***
***ABCG2*** ***Q141K*** ***rs2231142***	***---***	***0*.*1032/0*.*0944***	***0*.*0962***	***0*.*9371***	***1*.*02 (0*.*75–1*.*39)***	***0*.*5741***	***0*.*93 (0*.*74–1*.*17)***
***ATP2B4*** ***haplotype1*** ***rs1541252***	***---***	***0*.*1082/0*.*1024***	***0*.*1365***	***0*.*0252***	***1*.*39 (1*.*05–1*.*84)***	***0*.*0108***	***1*.*30 (1*.*07–1*.*59)***
***SLC2A1*** ***rs1385129***	***---***	***0*.*2120/0*.*2187***	***0*.*2032***	***0*.*4237***	***0*.*91 (0*.*73–1*.*14)***	***0*.*5267***	***0*.*95 (0*.*80–1*.*12)***
***RAVER1*** ***rs74956615***	***Critical COVID disease*** *[17]*	***0*.*0211/0*.*0298***	***0*.*0494***	***0*.*0387***	***1*.*67 (1*.*04–2*.*69)***	*<0*.0001*	***2*.*38 (1*.*71–5*.*41)***
***MUC5B*** ***rs35705950***	***Lower chance of COVID hospitalization*, *less severe disease*** *[32, 33]*	***0*.*0352/0*.*1074***	***0*.*0941***	***0*.*3565***	***0*.*86 (0*.*64–1*.*18)***	***<0*.*0001***	***2*.*85 (2*.*22–3*.*65)***
***UGT2A1*.*2A2*** ***rs7688383***	***Loss of smell/taste*** *[36]*	***0*.*3664/0*.*3857***	***0*.*4005***	***0*.*5364***	***1*.*06 (0*.*88–1*.*28)***	***0*.*0451***	***1*.*16 (1*.*00–1*.*33)***

The occurrence of the selected SNPs in the two COVID-19 severity groups was also examined in the recessive, dominant and additive genetic models. In the recessive model, the wild-type and the heterozygous cases were considered the same, and the effect was examined in patients homozygous for the minor variant (wt, heterozygous– 0, homozygous -1). In the dominant model, the effect of the heterozygous and homozygous cases together was examined, the wild type group was treated separately (wt-0, heterozygous, homozygous-1). In the additive model, logistic regression was applied, and the 3 genetic groups were examined separately, (0-wt, 1-heterozygous, 2-homozygous). Logistic regression (simple logistic regression–Prism 10, Graph Pad) was performed and odds ratios along with the 95% confidence interval for odds ratios were visualized.

**Ethics statement.** This project was carried out with the ethical permission of 24004-7/2021/EihO, issued on 7 May 2021, by the NNK Hungary—ETT TUKEB and 20800-6/2020/EÜIG, issued on 5 May 2020, by the NNK Hungary—ETT TUKEB. Informed consent was obtained from all the subjects involved in the study.

## Results

### 1. Clinical parameters—potential association with disease severity

We collected and analyzed data from 869 hospitalized COVID-19 patients. For the statistical analysis, disease severity was grouped into two main categories: 592 patients were regarded as moderate (originally labeled 195 mild and 397 moderate), and 277 patients were regarded as severe (originally labeled 141 severe and 136 critical). Among the examined patients, 465 were male and 404 were female. The mean age at the time of study for severe and moderate patients was 65 and 61 years, respectively. A total of 274 hospitalized patients had diabetes mellitus; among these patients, 18 had type I diabetes, 252 had type II diabetes (92%), and 594 did not have diabetes. Fifty-three patients had bronchial asthma, while 815 patients did not. A total of 168 patients reported a loss of taste, and 602 patients had no such symptoms. The loss of a sense of smell was reported by 164 patients, while no such symptoms were recorded in 605 patients. The overall analysis of the genetic parameters related to their Hardy-Weinberg distributions and the p values of Chi^2^ probe, are available from the authors upon request.

**Fig 1:**
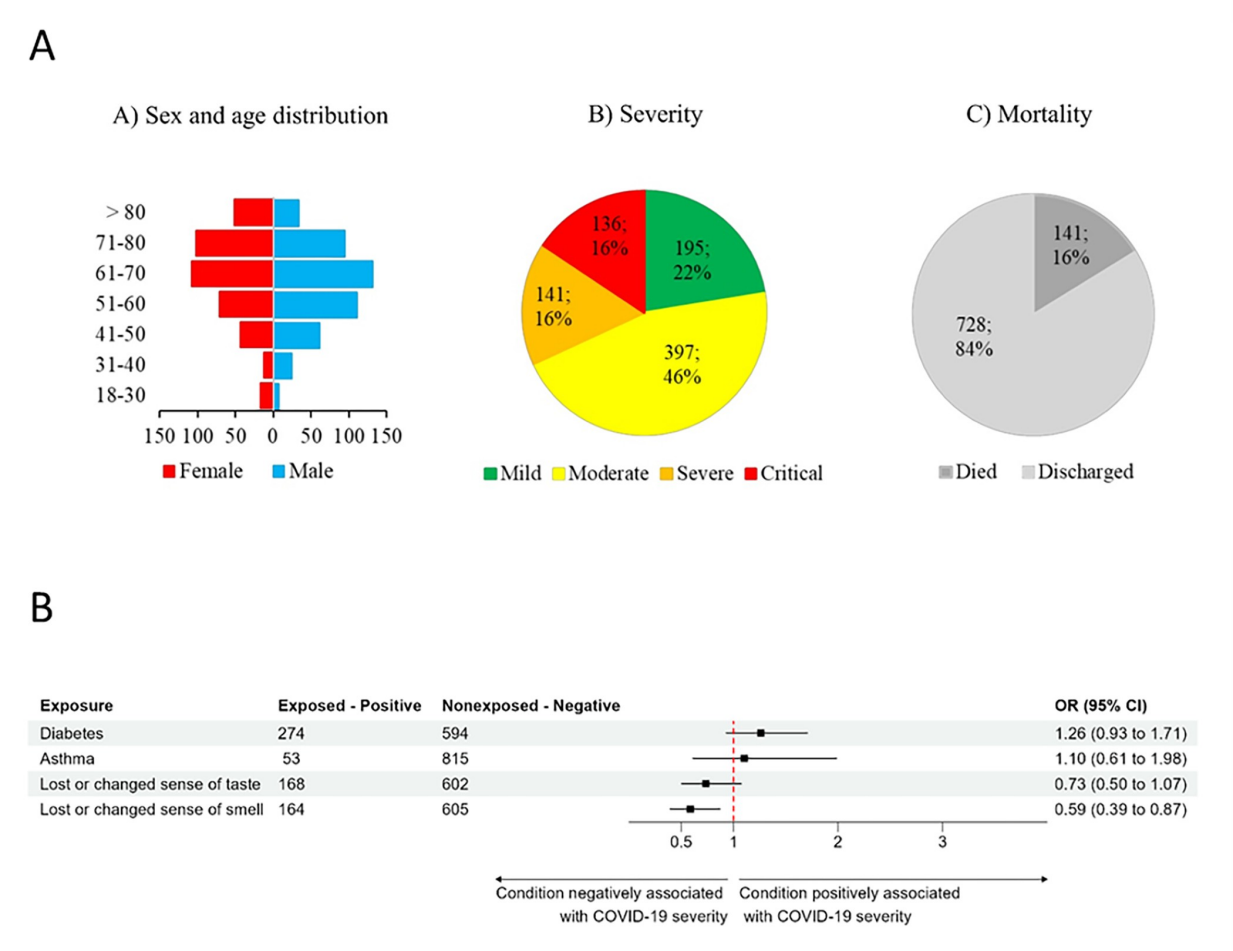
A. Summary of some key data for the population and disease severity of the COVID-19 patients included in this study. B. Associations of selected anamnestic parameters (categorical values) with disease severity in COVID-19 patients. The figure provides the number of positive and negative patients for each parameter and shows a forest plot for the odds ratios (ORs) for the associations of the selected parameters with disease severity. The forest plot indicates either lower average or higher average OR values and the respective 95% confidence intervals for severe, as compared to the mild COVID-19 cases.

The large amount of anamnestic and clinical data for COVID-19 patients treated at reporting clinics requires a further, detailed and systemic statistical analysis that correctly reflects the potential effects of these variables on the severity of the disease. These analyses are planned to be reported in a follow-up paper. Fig 1A represents a statistical analysis of the currently available data.

Fig 1B. shows the forest plot for some categorical clinical parameters with respect to disease severity. Here, we selected anamnestic parameters that have already been indicated in the relevant literature [1–4] to affect COVID-19 severity and had statistically acceptable numbers of patients in this study.

As indicated in Fig 1B, in accordance with the literature, we found a potential association between preexisting diabetes (in 92% of patients type 2 diabetes) and the severity of COVID-19 at the clinic. In contrast, we did not find a significant effect on disease severity in patients with asthma, while the potential effects of high blood pressure or cancer were not analyzed in this work. This lack of statistical significance may be caused by the relatively low number of patients suffering from these conditions. Interestingly, we found a significant association between the loss or altered sense of smell (and a similar tendency in the case of the loss or change of taste) and a less severe clinical form of the disease.

### 2. Potential associations of selected genetic polymorphisms with COVID-19 disease in hospitalized patients

In the first type of analysis, we examined the minor allele frequencies (MAFs) of the polymorphic variants of the selected genes in **all the hospitalized COVID-19 patients** and compared these MAF values to those in a **representative European population**. Significant differences should indicate the potential role of a given genetic polymorphism in the development of a COVID-19 disease (from mild to critical) requiring hospitalization.

Due to the lack of a proper control population (at the time of the data collection for our experiments no established tests or procedures were available to definitely rule out COVID-19 infection in a control group), our results are presented alongside the European MAF data from the widely used and accepted 1,000 Genomes Project and the ALFA database (see discussion). Due to occasional discrepancies in European MAF values between these two major sources, we included both sets in our analysis for a comprehensive evaluation. As shown in Table 1, we found that in several cases, the MAF values in the hospitalized patients showed major differences compared to the general European MAF values (in Table 1 we have also indicated previously reported COVID-19 disease associations and the respective references).

Although the two databases for the mean European MAF values are somewhat different (especially in the cases of *RAVER1* and *MUC5B*), these data together indicate that individuals carrying the minor variants of *IFNAR2 rs2236757*, *LZTFL1 rs73064425*, *RAVER1* rs74956615 and *ATP2B4 haplotype1-rs1541252*, may be generally more susceptible to hospitalization-requiring COVID-19 than the general population. In contrast, those carrying minor variants of the gene *OAS3* may be less likely to have a hospitalization-requiring disease.

In order to examine the potential differences in the European and Hungarian MAF values, respectively, in the case of the two most prominent indications for higher disease susceptibility *(IFNAR2 and LZTFL1*, we also measured these SNPs in a pre-COVID-19 DNA collection of 282 healthy Hungarian volunteers (reported in [41]). The MAF values obtained *(IFNAR2 rs2236757*: *0*.*29468 vs*. *EU MAF*: *0*.*2941/0*.*2942*, *and LZTFL1 rs73064425*: *0*.*103 –EU MAF*: *0*.*0820/0*.*0795*) in this relatively small sample showed a good correlation with the EU MAF values, indicating the validity of a significant difference for the hospitalized COVID-19 patients.

### 3. Potential associations of selected genetic polymorphisms with clinical severity of the COVID-19 disease

**Fig 2:**
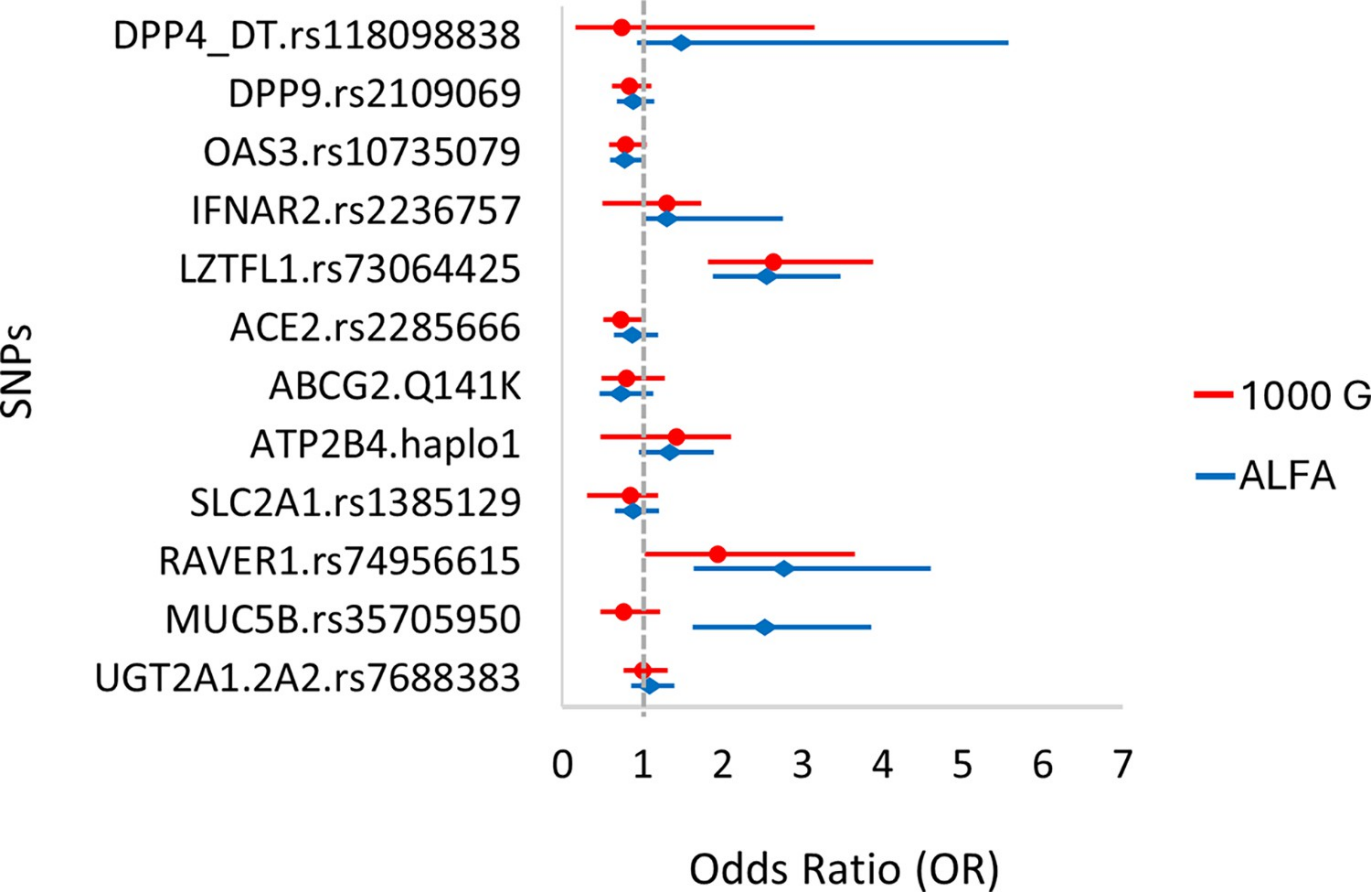
Allele frequencies of the minor variants (MAF) in the hospitalized COVID-19 patients with a severe disease, compared to the European MAF in the 1000 Genome (phase3 release V3+) and the ALFA (Release Version: 20230706150541) databases.

In the following we analyzed the potential involvement of the selected genetic polymorphisms **in COVID-19 disease severity.** In the first type of analysis, we compared the allele frequencies of the selected SNPs in the severe (clinically categorized as severe+critical) COVID-19 patients to the EU average allele frequencies. Again, we used both the 1000 Genome and ALFA databases for these comparisons. The results are presented in Fig 2 in the form of a forest plot for the calculated odds ratios (OR).

The data shown in Fig 2 (and detailed in S3 Table in S1 File) indicate that COVID-19 disease severity was significantly more prevalent in individuals carrying the minor variant of *LZTFL1*, and based on the ALFA database, also *IFNAR2*, *RAVER1*, *and MUC5B*. In contrast, those carrying minor variants of the gene *DPP9*, *OAS3*, *ACE2 and ABCG2* may be less likely to have severe disease among the hospitalized patients (although these differences do not reach a statistical significance).

**Fig 3:**
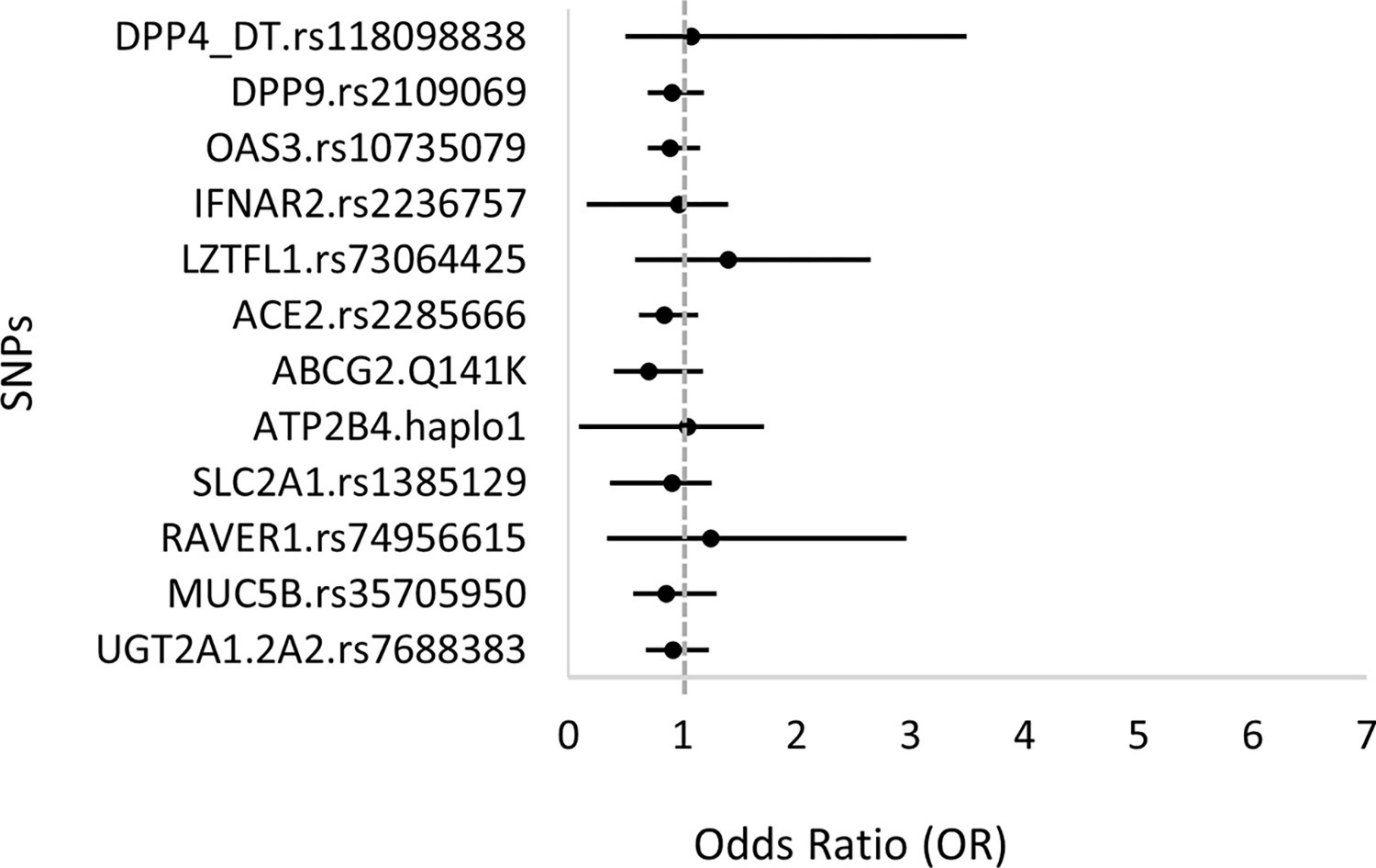
Comparison of the respective genetic variants between the groups of patients with mild+moderate, versus severe+critical COVID-19 disease. The odds ratios are shown for the **recessive genetic model** (two copies of the minor allele).

In the following we aimed for a direct analysis of the potential differences between the prevalence of the selected SNPs between the patients with less severe and severe disease. Due to the lack of a proper control population (see above), we used the combined mild/moderate groups of hospitalized patients to serve as the closest proxy for a control group, and used this mild/moderate patient group to compare the SNPs in the group of patients with severe (severe+critical) disease. Here we performed three types of analyses, corresponding to the recessive, dominant, or additive genetic models. These results are presented in Fig 3 and Supplementary materials, Figs + Tables 4 in S1 File, in the form of forest plots for the calculated ORs.

In the case of the tested *LZTFL1* SNP, both the additive and the recessive genetic models showed a significant association between the minor *LZTFL1* variant and disease severity. (see Fig 3 + detailed statistics in supplementary Figs and Tables 4 in S1 File). Although not statistically significant, an indication for a potential association of a more severe disease with the minor variants of *MUC5B* and *RAVER1* could be observed. These data also indicate (although with no statistical significance) that patients carrying a minor variant of *ABCG2* may be less likely to have a severe disease among the hospitalized patients. In the dominant genetic model, none of the SNPs showed association with disease severity, and the minor variant of *LZTFL1* showed an association in both the additive and recessive models, which indicates a robust association of this minor variant and COVID severity (see Supplementary Figs and Tables 4 in S1 File).

### 4. Potential association of genetic polymorphisms with preexisting type 2 diabetes mellitus (T2DM) in COVID-19 patients

According to the literature [3] and our data shown above (see Fig 1), the presence of diabetes in patients correlates with greater COVID-19 hospitalization. Therefore, we analyzed the potential associations of the examined genetic polymorphisms with the presence of diabetes and disease occurrence and severity.

First, we analyzed the associations of the minor variants of the polymorphisms in the 12 genes examined with COVID-19 severity in diabetic and nondiabetic patients. However, due to the relatively small number of patients, the confidence intervals for the potential associations of disease severity with preexisting diabetes and the examined genetic polymorphisms were large in most cases, and these differences did not reach statistical significance.

**Fig 4:**
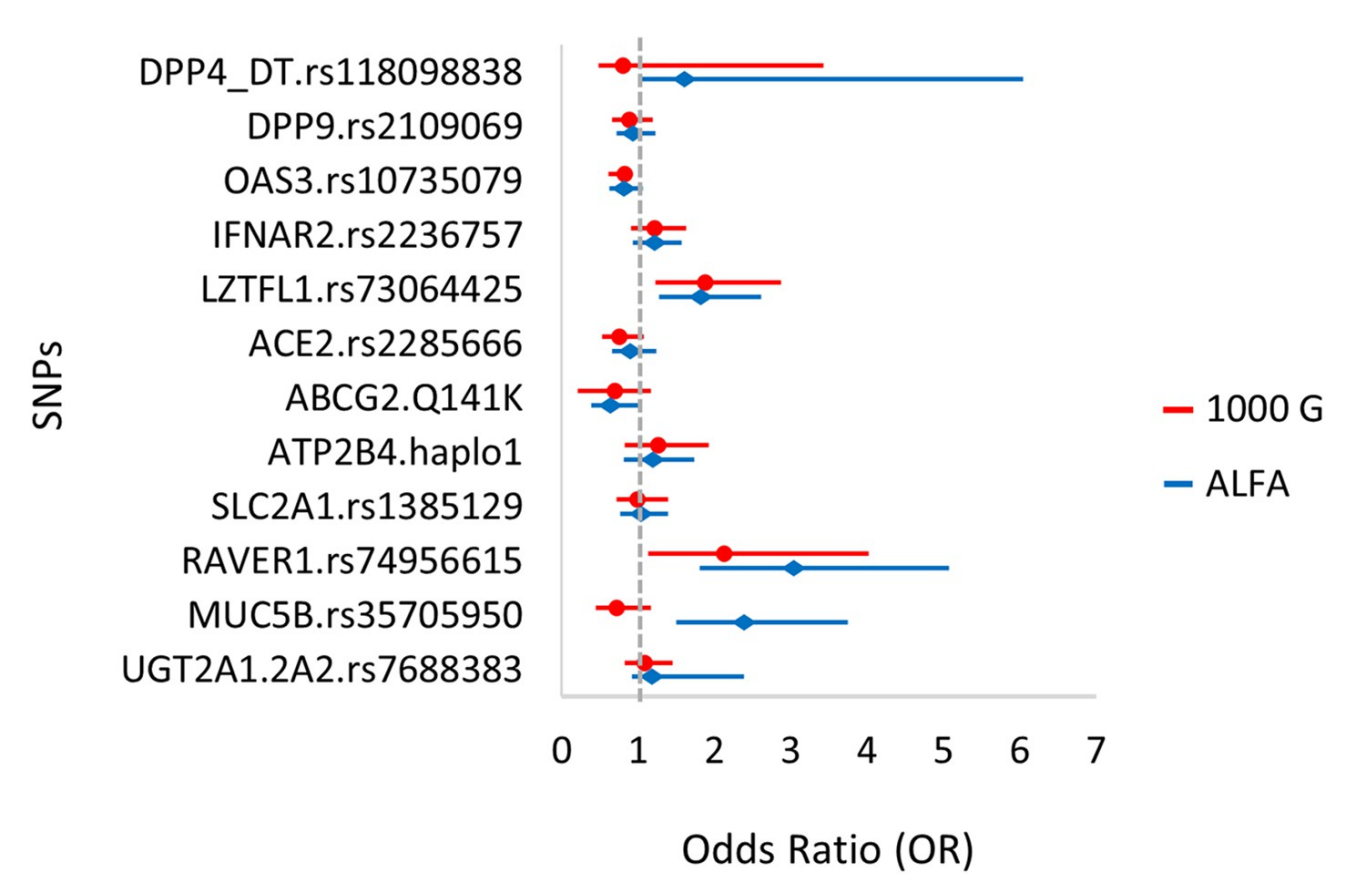
Allele frequencies of the minor variants (MAF) in the hospitalized COVID-19 patients with an anamnesis of type 2 diabetes, compared to the European MAF in the 1000 Genome (phase3 release V3+) and the ALFA (Release Version: 20230706150541) databases. This analysis included 252 diabetic patients.

As before, in order to examine the effects of the SNPs, we analyzed the MAF values of the polymorphic variants of the examined genes by comparing the diabetic hospitalized patients to the mean European minor allele frequencies (see Fig 4).

These data indicate that patients with type 2 diabetes carrying minor variants of *LZTFL1* and *RAVER1* (and probably also *IFNAR2* and *MUC5B)* are more likely to have hospitalization-requiring COVID-19 than the general population. In contrast, diabetic patients carrying the minor variant of *ABCG2*, are probably less likely to have hospitalization-requiring COVID-19. The odds ratio (OR) for the association of ABCG2 minor variant is around 0.6, while the association was not statistically significant (for the detailed statistics see Supplementary materials 5 in S1 File).

### 5. Potential association of genetic polymorphisms with the anamnestic parameters of loss of smell and/or taste or having asthma in patients with COVID-19

In the previous analysis, we found a significant association between lower disease severity and a reported loss of taste and/or smell (see Fig 1). In the following, we analyzed the potential associations of the examined genetic polymorphisms with these anamnestic parameters. Since these anamnestic parameters are variable in clinical reports, we analyzed them separately.

**Fig 5:**
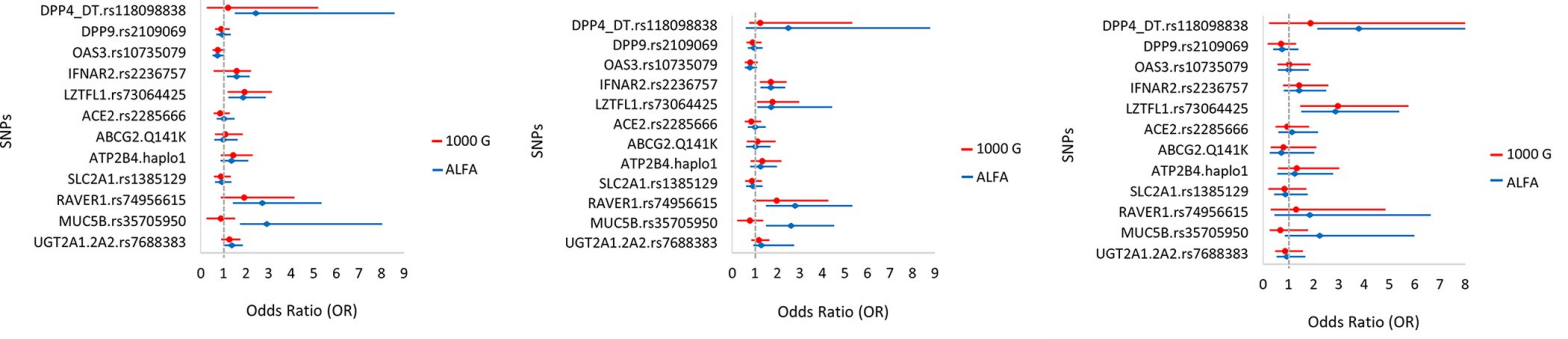
Allele frequencies of the minor variants (MAF) in the hospitalized COVID-19 patients with or without an anamnesis of loss of taste (Panel A), loss of smell (Panel B), or previous asthma (Panel C), compared to the European MAF in the 1000 Genome (phase3 release V3+) and the ALFA (Release Version: 20230706150541) databases. **Panel A:** *Loss of Taste—this analysis included 168 patients*. **Panel B.** *Loss of smell—this analysis included 164 patients*. **Panel C.** *Patients having asthma—this analysis included 32 patients*.

In these cases, we also analyzed the MAF values of the polymorphic variants of the examined genes in **hospitalized** patients reporting or not reporting a **loss of taste and/or smell,** respectively. We also included the analysis of patients **reporting asthma**, and compared these MAF values to the values in the representative European population (see Fig 5, and for the detailed statistics Supplementary materials, S6A-S6C Tables in S1 File).

These data collectively indicate similar correlations as those for the overall hospitalized patients: COVID-19 patients with loss of taste or smell, carrying the minor variants of IFNAR2, *LZTFL1* and *RAVER1* (and probably also *MUC5B* and *UGT2A1)*, are more likely to have hospitalization-requiring COVID-19 disease than the general population. In the case of patients having asthma, the only significant correlation was found for the presence of the *LZTFL1* minor variant and the hospitalization of these patients. In Supplementary material 7 in S1 File we document the distribution of the patients having diabetes and/or asthma according to disease severity groups.

When we directly examined the potential associations of the examined genetic polymorphisms with COVID-19 **disease severity** in patients reporting a loss of taste, a loss of sense of smell, or having asthma, due to the relatively small number of the respective patients, the confidence intervals for the potential associations of disease severity were large and the potential differences did not reach statistical significance. Also, none of the genetic models applied reached a significant conclusion for the role of the polymorphisms in these specific patient populations.

## Discussion

In the present work, we analyzed selected clinical and genetic data from 869 hospitalized COVID-19 patients in Hungary. Clinical data and blood samples were collected between 2020 and 2021, and the collection did not include effectively vaccinated patients. The dominant SARS-CoV-2 variants at that time were the Wuhan and the Delta forms. The detailed anamnestic and clinical laboratory data provided the opportunity to identify associations between these parameters, and for the genetic analyses, we selected the SNPs and the related haplotypes of 12 genes. Among the selected SNPs were those shown to be relevant to the reaction to COVID-19 according to genetic studies, including GWAS, in the literature [14–16]. Other specific variants were included because of their involvement in conditions that, according to our hypothesis, could be relevant in COVID-19. These were SNPs in the genes *ABCG2* (the SNP shown to play a role in gout susceptibility, and ADME (absorption, distribution, metabolism, and excretion) of drugs [42]), *PMCA4b* (the SNP protecting against malaria [43]), *SLC2A1* (SNP associated with impaired T cell recovery in antiretroviral-treated HIV patients [38]), *RAVER1* (SNP associated with type-2 diabetes and involvement in chronic COVID19 has also been shown [17]), and *UGT2A1/2A2* (SNPs associated with loss of taste and smell in COVID-19).

Based on the data shown in the Results section and in the Supplementary Materials, here we summarize the main conclusions of this study:

1. Regarding the anamnestic parameters, preexisting diabetes had a major effect on the severity of COVID-19 at the clinic, while no such effects were observed for asthma (potentially because of the relatively low number of relevant patients). Interestingly, we found a significant association between the loss or altered sense of smell or taste and a less severe clinical form of COVID-19.

2. When looking for potential associations between the genetic variants in the 12 genes studied, we performed several types of analyses. First, we compared the minor allele frequency values in clinically treated COVID-19 patients to the MAF values in the general European population. We performed a similar analysis in patients with different disease severities (mild or severe) or preexisting conditions (comorbidity, loss of taste or smell). It is important to note that the Hungarian population in all aspects closely reflects the European genetic SNP patterns (see refs [44–46]), thus the MAF datasets for European population, provided by the 1000 Genome (phase3 release V3+) and the ALFA (Release Version: 20230706150541) databases for assessing population-based genetic differences. Although these databases in some cases provide variable data (see Table 1), they are still more relevant for such an analysis than a few hundred control MAF values obtained locally. In case of two SNPs with observed disease significance *(IFNAR2 and LZTFL1)*, we examined the potential differences in the European and Hungarian MAF values, respectively, and found a close correlation of these values (see Results).

Another way of analyzing the associations of genetic factors with disease conditions was to directly compare the presence of minor allele variants in the categories of different disease severities or key anamnestic parameters. The comparison was made to less severe disease group in case of severity, and in other cases to the standard minor allele variant frequencies in the general European population. According to these combined analyses, the following conclusions can be drawn:

a. Regarding the **hospitalized COVID-19 patients**, higher minor allele frequencies (MAF) of the *IFNAR2* rs2236757 (OR 1.33), *LZTFL1* rs73064425, (OR 2.04–2.11), *RAVER1* rs74956615 (OR 1.7–2.4), and potentially *MUC5B* rs35705950 (OR regarding the ALFA database 2.85) significantly correlated with greater COVID-19 disease susceptibility. The strong effect of *LZTFL1* on COVID-19 susceptibility is in accordance with the data in the literature (see refs [10–14, 17–24, 27]), while the effect of the *IFNAR2* minor variant has been variably observed [28, 47, 48]. In contrast to some of the data in the literature [17], the *RAVER1* rs74956615 minor allele was more common in the COVID-19 patients than the EU average. If the mild+moderate group with the severe+critical COVID-19 illness group was compared, the same tendency could be observed (although not statistically significant, this SNP was more common among the severe patients). *MUC5B* rs35705950 in one report was found to be protective in terms of COVID-19 hospitalization [32], but not severity, and it was associated with less severe disease according to another publication [33], although carriers of the minor variant are known to be more susceptible to idiopathic pulmonary fibrosis [49]. Our results suggest a positive correlation of this variant with COVID-19 hospitalization.

Interestingly, the MAF value of the *ATP2B4* haplotype 1 (examined SNP: rs1541252) minor variant was found to be higher in the hospitalized patients than in the general population. This new observation may indicate an increased COVID-19 disease susceptibility (OR 1.39–1.30) in the presence of this variant, shown to be protective in malaria [43]. The SNPs in *DPP4-DT* (probably because of the low MAF values and the relatively small population), *DPP9*, *ABCG2*, and *SLC2A1* did not show significant associations with overall COVID-19-related hospitalization in our analysis.

b. When examining the associations of genetic variations with the **severity of COVID-19** disease, we have used the EU MAF data for comparison (Fig 2), and also applied three different genetic models (recessive, dominant and additive–see Fig 3 and Supplement 4 in S1 File) for a direct analysis. In the latter case we used the combination of patients with mild and moderate clinical disease as controls versus the severe clinical forms. We observed that a higher frequency of the *LZTFL1* minor variant associated with greater disease severity, and a similar tendency was observed in the cases of the *RAVER1*, *MUC5B*, and *UGT2A1* variants (significant only when compared to the ALFA population values). The direct analysis in the recessive and additive genetic models indicated similar tendencies, while there were no significant associations found in the dominant model. Our findings align with previous studies that have linked the minor T allele of rs73064425 in the *LZTFL1* gene to an increased risk of severe COVID-19 outcomes. This specific variant has been associated with enhanced susceptibility to SARS-CoV-2 infection and more severe disease progression [24, 50]. Mechanistically, rs73064425 has been implicated in the upregulation of *LZTFL1* expression, which modulates epithelial-mesenchymal transition (EMT) in pulmonary epithelial cells—a pathway involved in immune response and tissue repair during lung infections [18]. This dysregulated EMT may contribute to severe respiratory damage seen in COVID-19 [51], underscoring the importance of *LZTFL1* as a potential therapeutic target for mitigating COVID-19 severity.

c. In COVID-19 patients who also had type 2 diabetes, our results indicated that patients carrying the minor variants of *LZTFL1* and *RAVER1* (and probably also *IFNAR2* and *MUC5B)* are more likely to have hospitalization-requiring COVID-19 than the general population.

In contrast, we observed that type 2 diabetic patients carrying the minor variant of *ABCG2*, are probably less likely to have hospitalization-requiring COVID-19, although these results were not statistically significant (see Fig 4). Thus, the presence of the rather common genetic variant of the ABCG2 multidrug transporter (SNP rs2231142, Q141K amino acid change) seems to be associated with a protective effect in diabetic patients. As this frequent variant has been shown to have a role in the development of gout [52, 53] and in the pharmacokinetics of a wide range of commonly used drugs [54], further exploration of this genetic association should be performed in a larger patient population and potentially in patients with long-term COVID-19. In our studies, in patients reporting a loss of smell or taste, the examined genetic associations showed a pattern similar to that in the general hospitalized COVID-19 population.

When disease-related SNPs are identified through GWAS, these variables often have only small effects on the respective ORs and may be lost in the background noise. By using high-level statistical thresholds, only genetic variants with strong statistical evidence can be regarded as significant; thus, variants with smaller effects are filtered out. Especially in infectious diseases with variable tissue pathologies, multiple genetic variants with small individual effects may contribute to the overall risk. Therefore, although our current study focused on a limited number of potentially relevant genetic variants in a relatively small number of patients, it may enable a targeted analysis of clinical and genetic data with potential relevance to the COVID-19 clinic.

While the study’s limited scope might restrict broad conclusions, the data in this study may help to decipher the association between selected genetic variants and the incidence of COVID-19. The data were obtained for people infected with the Wuhan and Delta variants; thus, they may not be relevant to the clinical effects of the newly emerging SARS-CoV-2 variants, e.g., the Omicron variants. Nevertheless, the basic clinical problems in severe cases of current diseases involve the same cellular virus receptors and the overreaction of the immune system (see [55, 56]). Most of the genetic variations studied here are related to virus receptors, the virus-activated immune system, or general metabolic regulators.

A newly emerging question is the potential genetic background of the rapidly increasing number of long-term or post-COVID-19 patients with currently ill-defined clinical symptoms (see [57–63]). When extended to the current COVID-19 situation and using larger patient numbers, our approach may help to provide a personalized tool for assessing the expected course, severity, and long-term, chronic effects in COVID-19 patients.

### Limitations of the study

The present report acknowledges several limitations of this study. The first one is the absence of a large cohort of Hungarian control samples. However, at the time of our data collection, no proper tests or procedures were available to rule out COVID-19 infection, thereby preventing the establishment of a related control group. Consequently, our results are presented for the hospitalized COVID-19 patients in comparison with the genetic data from the widely utilized 1000 Genomes Project and the ALFA database. Second, a population stratification has not been performed in our study, due to the limited data availability in this regard.

## Supporting information

S1 File Supporting information to this paper can be accessed at the file: Supplementary Materials for Mózner et al: Potential correlation of selected polymorphic genetic variants with COVID-19 disease susceptibility and severity. (PDF)
