# Supplementary material for: Potential associations of selected polymorphic genetic variants with COVID-19 disease susceptibility and severity
Source: PLoS One. 2025 Jan 3;20(1):e0316396. doi: 10.1371/journal.pone.0316396 (PMC11698323; doi:10.1371/journal.pone.0316396)

Supplementary Materials for Mózner et al: Potential correlation of selected polymorphic genetic variants with COVID-19 disease susceptibility and severity

Supplementary table 1: SNPs analyzed and their minor allele frequency (MAF) in the European population. MAF (Eur) from the dbSNP databases (see **https://www.ncbi.nlm.nih.gov/snp/)**

SNP	MAF (Eur) (ALFA/1000G)	Related gene(s)	COVID-19 or other disease association	References
rs118098838 (chr 2)	0.0050/ 0.0099	*DPP4-DT,* NRP1 and NRP2	Severe COVID-19 symptoms	[1]
rs2109069 (chr 12)	0.3088/ 0.3211	*DPP9*	Severe COVID-19 symptoms	[1]
rs10735079 (chr 12)	0.3662/ 0.3638	*OAS3*	More/less severe COVID-19 symptoms	[1]
rs2236757 (chr 21)	0.2941/ 0.2942	*IFNAR2*	Severe COVID-19 symptoms	[2–4]
rs73064425 (chr 3)	0.0820/ 0.0795	*LZTFL1*	Severe COVID-19 symptoms	[5,6]
rs2285666 (chr X)	0.2038/ 0.2350	*ACE2*	Affects ACE2 protein expression, lower COVID-19 infection	[7]
rs2231142 (chr 4)	0.1032/ 0.0944	*ABCG2*	Decreased ABCG2 protein expression, gout susceptibility	[8]
rs1541252 (chr 1)	0.1082/ 0.1024	*ATP2B4*	Regulation of PMCA4b expression	[9,10]
rs1385129 (chr 1)	0.2120/ 0.2187	*SLC2A1*	Poor CD4+ T cell recovery in antiretroviral-treated individuals	[11]
rs74956615 (chr 19)	0.0211/ 0.0298	*RAVER1*	Associated with critical illness in COVID-19	[12]
rs35705950 (chr 11)	0.0352/ 0.1074	*MUC5B*	Associated with less severe COVID-19	[13,14]
rs7688383 (chr 4)	0.3664/ 0.3857	*UGT2A1*	Associated with loss of smell or taste in COVID-19	[15]

References for Supplementary table 1

1. Pairo-Castineira E, Clohisey S, Klaric L, Bretherick AD, Rawlik K, Pasko D, et al. Genetic mechanisms of critical illness in COVID-19. Nature. 2021;591: 92–98. doi:10.1038/s41586-020-03065-y

2. Yaugel-Novoa M, Bourlet T, Longet S, Botelho-Nevers E, Paul S. Association of IFNAR1 and IFNAR2 with COVID-19 severity. Lancet Microbe. 2023;4: e487. doi:10.1016/S2666-5247(23)00095-2

3. López-Bielma MF, Falfán-Valencia R, Abarca-Rojano E, Pérez-Rubio G. Participation of Single-Nucleotide Variants in IFNAR1 and IFNAR2 in the Immune Response against SARS-CoV-2 Infection: A Systematic Review. Pathogens. 2023;12. doi:10.3390/pathogens12111320

4. Fricke-Galindo I, Martínez-Morales A, Chávez-Galán L, Ocaña-Guzmán R, Buendía-Roldán I, Pérez-Rubio G, et al. IFNAR2 relevance in the clinical outcome of individuals with severe COVID-19. Front Immunol. 2022;13: 949413. doi:10.3389/fimmu.2022.949413

5. Downes DJ, Cross AR, Hua P, Roberts N, Schwessinger R, Cutler AJ, et al. Identification of LZTFL1 as a candidate effector gene at a COVID-19 risk locus. Nat Genet. 2021;53: 1606–1615. doi:10.1038/s41588-021-00955-3

6. Rüter J, Pallerla SR, Meyer CG, Casadei N, Sonnabend M, Peter S, et al. Host genetic loci LZTFL1 and CCL2 associated with SARS-CoV-2 infection and severity of COVID-19. Int J Infect Dis. 2022;122: 427–436. doi:10.1016/j.ijid.2022.06.030

7. Srivastava A, Bandopadhyay A, Das D, Pandey RK, Singh V, Khanam N, et al. Genetic Association of ACE2 rs2285666 Polymorphism With COVID-19 Spatial Distribution in India. Front Genet. 2020;11: 564741. doi:10.3389/fgene.2020.564741

8. Hoque KM, Dixon EE, Lewis RM, Allan J, Gamble GD, Phipps-Green AJ, et al. The ABCG2 Q141K hyperuricemia and gout associated variant illuminates the physiology of human urate excretion. Nat Commun. 2020;11: 2767. doi:10.1038/s41467-020-16525-w

9. Lessard S, Gatof ES, Beaudoin M, Schupp PG, Sher F, Ali A, et al. An erythroid-specific ATP2B4 enhancer mediates red blood cell hydration and malaria susceptibility. Journal of Clinical Investigation. 2017;127: 3065–3074. doi:10.1172/JCI94378

10. Zámbó B, Várady G, Padányi R, Szabó E, Németh A, Langó T, et al. Decreased calcium pump expression in human erythrocytes is connected to a minor haplotype in the ATP2B4 gene. Cell Calcium. 2017;65: 73–79. doi:10.1016/j.ceca.2017.02.001

11. Masson JJR, Cherry CL, Murphy NM, Sada-Ovalle I, Hussain T, Palchaudhuri R, et al. Polymorphism rs1385129 Within Glut1 Gene SLC2A1 Is Linked to Poor CD4+ T Cell Recovery in Antiretroviral-Treated HIV+ Individuals. Front Immunol. 2018;9: 900. doi:10.3389/fimmu.2018.00900

12. Fink-Baldauf IM, Stuart WD, Brewington JJ, Guo M, Maeda Y. CRISPRi links COVID-19 GWAS loci to LZTFL1 and RAVER1. EBioMedicine. 2022;75: 103806. doi:10.1016/j.ebiom.2021.103806

13. Verma A, Minnier J, Wan ES, Huffman JE, Gao L, Joseph J, et al. A MUC5B Gene Polymorphism, rs35705950-T, Confers Protective Effects Against COVID-19 Hospitalization but Not Severe Disease or Mortality. Am J Respir Crit Care Med. 2022;206: 1220–1229. doi:10.1164/rccm.202109-2166OC

14. van Moorsel CHM, van der Vis JJ, Duckworth A, Scotton CJ, Benschop C, Ellinghaus D, et al. The MUC5B Promoter Polymorphism Associates With Severe COVID-19 in the European Population. Front Med (Lausanne). 2021;8: 668024. doi:10.3389/fmed.2021.668024

15. Shelton JF, Shastri AJ, Fletez-Brant K, 23andMe COVID-19 Team, Aslibekyan S, Auton A. The UGT2A1/UGT2A2 locus is associated with COVID-19-related loss of smell or taste. Nat Genet. 2022;54: 121–124. doi:10.1038/s41588-021-00986-w

Supplementary table 2: SNPs and DNA sequences of the probes used in qPCR experiments

SNPs	Order ID	**Context Sequence [VIC/FAM]**
rs118098838	C_150493403_10	CCTTTATCATATAGATACAATTTTC[G/T]CTTAATAATAGCATGCTCCTGCAAA
rs2109069	C__11517118_10	TCACCCAGAGAGGAAGGGGAGTGGA[G/A]CCCCAGTCTCTTGGAGCCCAAAACC
rs10735079	C__31831768_10	TGGCTAGCAGTAGGGGCCTGGGGAC[A/G]AAACCAGAATTCTGCAAAATCTTGT
rs2236757	C__11354003_30	CAAATCCCAAAAGAGATTAAGGCCT[A/G]CCTCTAAATGAAATTCTCAGTCTTA
rs73064425	C__98755833_10	CTCATTTTTAAATGACAAAAATTAA[C/T]GAATGAATGAAAGTGGATCTTTCAC
rs2285666	C___2551626_1_	ATAATCACTACTAAAAATTAGTAGC[C/T]TACCTGGTTCAAGTAATAAGCATTC
rs1541252	C____360682_10	TCCTCTTCCTCCTCCTGACGTCTAC[C/T]ACTACAGTTGCTGGTTGTTGCTAAG
rs1385129	C___1166185_1_	GGGAGCCAAGCACTGCTCCTCCCAC[A/G]GCCAGCATGAGGCGACCCGTCAGCT
rs2231142	C__15854163_70	GCAAGCCGAAGAGCTGCTGAGAACT[G/T]TAAGTTTTCTCTCACCGTCAGAGTG
rs74956615	C__27854626_20	TAGAAAAGGAAACAGAAGTCAGTTG[T/A]CAAAGTTAAAAAAAAAGGAGACAGT
rs35705950	C___1582254_20	CCTTCCTTTATCTTCTGTTTTCAGC[G/T]CCTTCAACTGTGAAGAGGTGAACTC
rs7688383	C___2951323_10	TTTATTGCAGTACTGTTCCCAATAG[C/T]CAAGATATGGAATCAACCTATATGT

Supplementary table 3: *Odds ratio analysis in the hospitalized COVID-19 patients with a severe disease, compared to the European MAF in the 1000 Genome (phase3 release V3+) and the ALFA (Release Version: 20230706150541) databases. Statistical analysis was performed by Fisher test with Odds ratio analysis. In all cases the Bonferroni corrections were also performed (see manuscript).*

Gene polymorphism	*Ref.: 1000Genomes (EUR)*			*Ref.: ALFA (EUR)*		
	p value	*p value (Bonferroni correction)*	*OR (95% CI)*	*p value*	*p value (Bonferroni correction)*	*OR (95% CI)*
DPP4_DT rs118098838	>0.9999	>0.9999	0.74 (0.16-3.15)	0.3964	>0.9999	1.49 (0.92-5.57)
DPP9 rs2109069	0.2412	>0.9999	0.83 (0.62-1.12)	0.3621	>0.9999	0.88 (0.68-1.15)
OAS3 rs10735079	0.0999	>0.9999	0.78 (0.59-1.05)	0.0509	0.6108	0.77 (0.60-1.00)
IFNAR2 rs2236757	0.0654	0.7848	1.31 (0.49-1.74)	0.0393	0.4716	1.31 (1.02-2.75)
LZTFL1 rs73064425	<0.0001	<0.0012	2.64 (1.82-9.88)	<0.0001	<0.0012	2.55 (1.88-3.47)
ACE2 rs2285666	0.0893	>0.9999	0.73 (0.51-1.03)	0.4517	>0.9999	0.88 (0.64-1.19)
ABCG2 rs2231142	0.4072	>0.9999	0.79 (0.49-1.27)	0.1642	>0.9999	0.72 (0.46-1.13)
ATP2B4 rs1541252	0.1011	>0.9999	1.42 (0.48-2.10)	0.0968	>0.9999	1.34 (0.95-1.89)
SLC2A1 rs1385129	0.3993	>0.9999	0.85 (0.30-1.20)	0.4983	>0.9999	0.89 (0.65-1.20)
RAVER1 rs74956615	0.0596	0.7152	1.94 (1.03-3.65)	0.0008	0.0096	2.76 (1.64-4.60)
MUC5B rs35705950	0.3111	>0.9999	0.77 (0.48-1.21)	0.0002	0.0024	2.53 (1.63-3.86)
UGT2A1.2A2 rs7688383	>0.9999	>0.9999	1.01 (0.77-1.32)	0.4868	>0.9999	1.09 (0.86-1.40)

Supplementary Figures and Tables 4. Genetic analysis of the minor alleles by using three different models

4A. Recessive genetic model

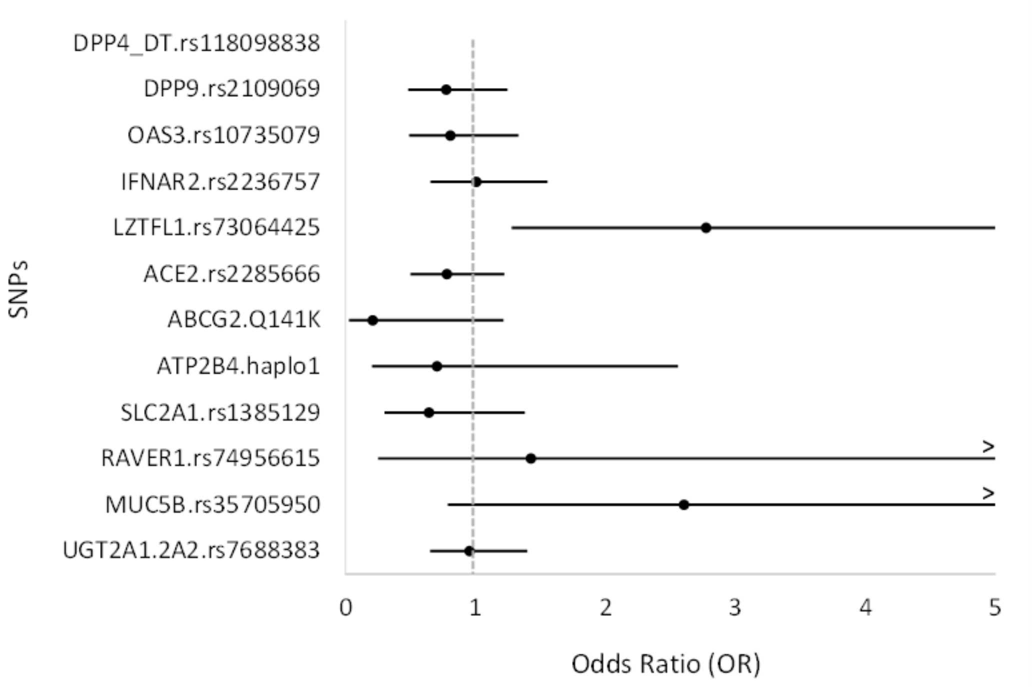

Gene polymorphism	***Ref.: patients with mild and moderate COVID-severity***	
	p value	***OR (95% CI)***
DPP4_DT rs118098838	***>0.9999***	
DPP9 rs2109069	***0.3445***	***0.77 (0.48-1.25)***
OAS3 rs10735079	***0.4647***	***0.81 (0.49-1.33)***
IFNAR2 rs2236757	***>0,9999***	***1.01 (0.65-1.56)***
LZTFL1 rs73064425	***0.0107***	***2.77 (1.28-5.97)***
ACE2 rs2285666	***0.2815***	***0.78 (0.50-1.22)***
ABCG2 rs2231142	***0.1887***	***0.21 (0.03-1.22)***
ATP2B4 rs1541252	***0.761***	***0.71 (0.20-2.56)***
SLC2A1 rs1385129	***0.2924***	***0.64 (0.30-1.38)***
RAVER1 rs74956615	***0.6565***	***1.43 (0.25-7.02)***
MUC5B rs35705950	***0.1136***	***2.61 (0.79-7.51)***
UGT2A1.2A2 rs7688383	***0.8468***	***0.96 (0.65-1.40)***

4B. Dominant genetic model

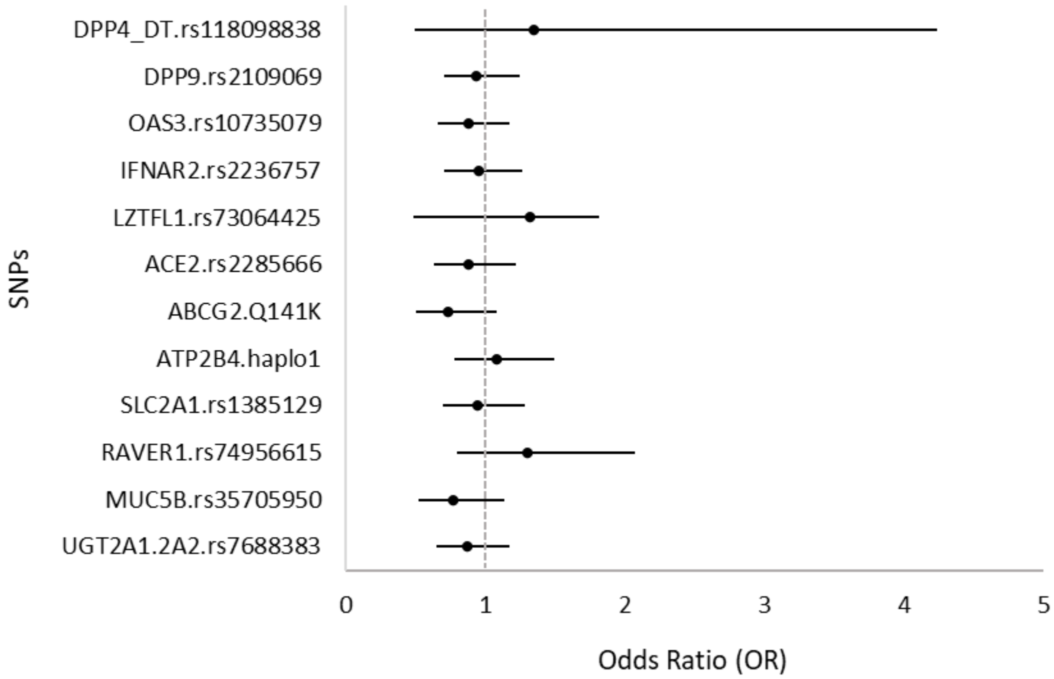

Gene polymorphism	*Ref.: patients with mild and moderate COVID-severity*	
	p value	*OR (95% CI)*
DPP4_DT rs118098838	***0.5638***	***1.35 (0.49-4.23)***
DPP9 rs2109069	***0.6618***	***0.94 (0.70-1.25)***
OAS3 rs10735079	***0.4171***	***0.88 (0.66-1.17)***
IFNAR2 rs2236757	***0.7668***	***0.95 (0.71-1.26)***
LZTFL1 rs73064425	***0.0873***	***1.32 (0.48-1.81)***
ACE2 rs2285666	***0.4601***	***0.88 (0.63-1.21)***
ABCG2 rs2231142	***0.1277***	***0.73 (0.50-1.01)***
ATP2B4 rs1541252	***0.6752***	***1.08 (0.77-1.49)***
SLC2A1 rs1385129	***0.7574***	***0.94 (0.69-1.28)***
RAVER1 rs74956615	***0.3087***	***1.30 (0.79-2.07)***
MUC5B rs35705950	***0.1798***	***0.76 (0.53-1.13)***
UGT2A1.2A2 rs7688383	***0.3636***	***0.87 (0.65-1.17)***

4C. Additive genetic model

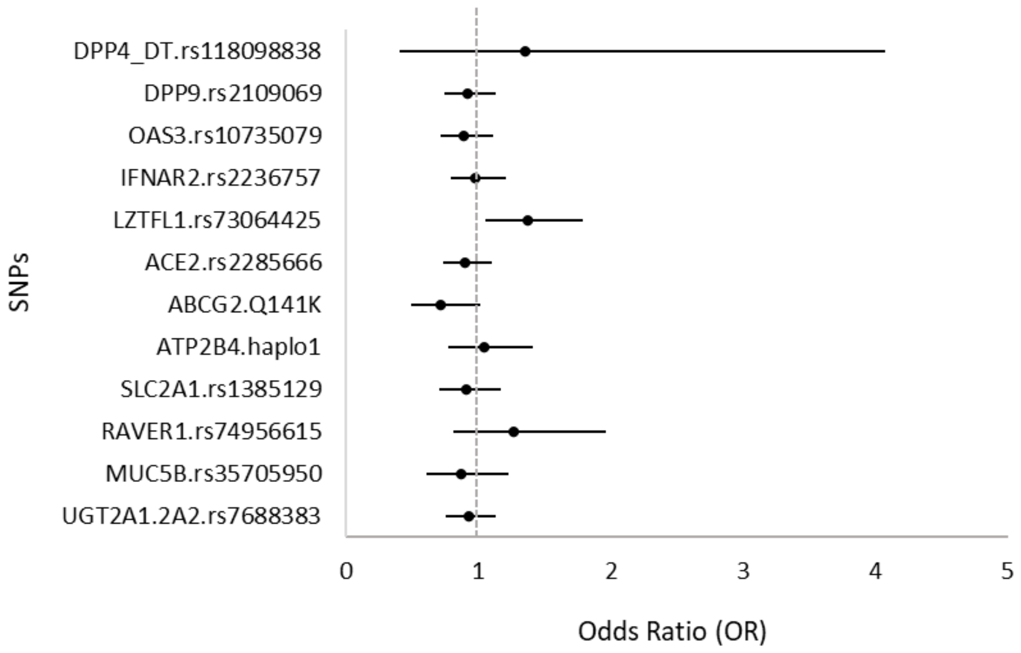

Gene polymorphism	*Ref.: patients with mild and moderate COVID-severity*	
	p value	*OR (95% CI)*
DPP4_DT rs118098838	***0.6035***	***1.35 (0.40-4.08)***
DPP9 rs2109069	***0.4136***	***0.91 (0.74-1.13)***
OAS3 rs10735079	***0.2931***	***0.89 (0.71-1.11)***
IFNAR2 rs2236757	***0.8016***	***0.97 (0.79-1.20)***
LZTFL1 rs73064425	***0.0186***	***1.37 (1.05-1.79)***
ACE2 rs2285666	***0.3120***	***0.90 (0.73-1.10)***
ABCG2 rs2231142	***0.0626***	***0.71 (0.49-1.01)***
ATP2B4 rs1541252	***0.7858***	***1.04 (0.77-1.41)***
SLC2A1 rs1385129	***0.4648***	***0.91 (0.70-1.17)***
RAVER1 rs74956615	***0.2939***	***1.27 (0.81-1.96)***
MUC5B rs35705950	***0.4305***	***0.87 (0.61-1.22)***
UGT2A1.2A2 rs7688383	***0.4422***	***0.92 (0.75-1.13)***

Supplementary material 5: *Odds ratio analysis of patients with type 2 diabetes mellitus, compared to the European MAF in the 1000 Genome (phase3 release V3+) and the ALFA (Release Version: 20230706150541) databases. Statistical analysis was performed by Fisher test with Odds ratio analysis. In all cases the Bonferroni corrections were also performed.*

Gene polymorphism	Ref.: 1000Genomes (EUR)			Ref.: ALFA (EUR)		
	p value	p value (Bonferroni correction)	OR (95% CI)	p value	p value (Bonferroni correction)	OR (95% CI)
DPP4_DT rs118098838	>0.9999	>0.9999	0.80 (0.49-3.42)	0.3594	>0.9999	1.61 (1.00-6.05)
DPP9 rs2109069	0.4484	>0.9999	0.88 (0.66-1.19)	0.6818	>0.9999	0.94 (0.71-1.23)
OAS3 rs10735079	0.2097	>0.9999	0.82 (0.61-0.61)	0.1314	>0.9999	0.81 (0.62-1.06)
IFNAR2 rs2236757	0.2183	>0.9999	1.21 (0.91-1.63)	0.1636	>0.9999	1.21 (0.93-1.58)
LZTFL1 rs73064425	0.0046	0.0552	1.88 (1.23-2.87)	0.0024	0.0288	1.82 (1.27-2.62)
ACE2 rs2285666	0.1374	>0.9999	0.75 (0.52-1.08)	0.5828	>0.9999	0.90 (0.66-1.24)
ABCG2 rs2231142	0.2156	>0.9999	0.70 (0.2-1.17)	0.0759	0.9108	0.64 (0.39-1.04)
ATP2B4 rs1541252	0.3003	>0.9999	1.26 (0.83-1.92)	0.3554	>0.9999	1.19 (0.82-1.73)
SLC2A1 rs1385129	>0.9999	>0.9999	0.99 (0.71-1.39)	0.8158	>0.9999	1.03 (0.76-1.40)
RAVER1 rs74956615	0.0326	0.3912	2.13 (1.13-4.03)	0.0003	0.0036	3.04 (1.81-5.08)
MUC5B rs35705950	0.2424	>0.9999	0.72 (0.44-1.17)	0.0008	0.0096	2.39 (1.50-3.75)
UGT2A1.2A2 rs7688383	0.5632	>0.9999	1.09 (0.82-1.45)	0.2093	>0.9999	1.18 (0.92-2.39)

Supplementary materials, table 6A: *Odds ratio analysis in the hospitalized COVID-19 patients with loss os taste, compared to the European MAF in the 1000 Genome (phase3 release V3+) and the ALFA (Release Version: 20230706150541) databases. Statistical analysis was performed by Fisher test with Odds ratio analysis. In all cases the Bonferroni corrections were also performed.*

Gene polymorphism	Ref.: 1000Genomes (EUR)			Ref.: ALFA (EUR)		
	p value	p value (Bonferroni correction)	OR (95% CI)	p value	p value (Bonferroni correction)	OR (95% CI)
DPP4_DT rs118098838	0.6841	>0.9999	1.21 (0.26-5.20)	0.2059	>0.9999	2.431 (1.51-8.58)
DPP9 rs2109069	0.6538	>0.9999	0.90 (0.64-1.28)	0.867	>0.9999	0.96 (0.69-1.33)
OAS3 rs10735079	0.1336	>0.9999	0.75 (0.53-1.07)	0.0877	>0.9999	0.74 (0.53-1.04)
IFNAR2 rs2236757	0.0086	0.1032	1.58 (0.56-2.22)	0.0047	0.0564	1.58 (1.16-2.16)
LZTFL1 rs73064425	0.0116	0.1392	1.94 (1.19-3.15)	0.0069	0.0828	1.88 (1.22-2.88)
ACE2 rs2285666	0.4758	>0.9999	0.85 (0.56-1.28)	0.9227	>0.9999	1.02 (0.70-1.49)
ABCG2 rs2231142	0.7761	>0.9999	1.09 (0.63-1.85)	>0.9999	>0.9999	0.99 (0.60-1.64)
ATP2B4 rs1541252	0.1364	>0.9999	1.44 (0.89-2.30)	0.1667	>0.9999	1.35 (0.87-2.11)
SLC2A1 rs1385129	0.6831	>0.9999	0.89 (0.59-1.33)	0.7752	>0.9999	0.93 (0.63-1.36)
RAVER1 rs74956615	0.0988	>0.9999	1.91 (0.89-4.15)	0.0084	0.1008	2.73 (1.42-5.36)
MUC5B rs35705950	0.7855	>0.9999	0.89 (0.26-1.53)	0.0003	0.0036	2.93 (1.73-8.04)
UGT2A1.2A2 rs7688383	0.1700	>0.9999	1.26 (0.90-1.75)	0.509	>0.9999	1.37 (1.01-1.87)

Supplementary table 6B: *Odds ratio analysis in the hospitalized COVID-19 patients with loss os smell, compared to the European MAF in the 1000 Genome (phase3 release V3+) and the ALFA (Release Version: 20230706150541) databases. Statistical analysis was performed by Fisher test with Odds ratio analysis. In all cases the Bonferroni corrections were also performed.*

Gene polymorphism	Ref.: 1000Genomes (EUR)			Ref.: ALFA (EUR)		
	p value	p value (Bonferroni correction)	OR (95% CI)	p value	p value (Bonferroni correction)	OR (95% CI)
DPP4_DT rs118098838	0.6787	>0.9999	1.24 (0.75-5.33)	0.1987	>0.9999	2.49 (0.66-8.79)
DPP9 rs2109069	0.6508	>0.9999	0.91 (0.63-1.30)	0.8656	>0.9999	0.96 (0.69-1.35)
OAS3 rs10735079	0.2150	>0.9999	0.79 (0.56-1.13)	0.1637	>0.9999	0.79 (0.56-1.10)
IFNAR2 rs2236757	0.0025	0.0300	1.72 (1.22-2.41)	0.0010	0.012	1.72 (1.26-2.35)
LZTFL1 rs73064425	0.0352	0.4224	1.79 (1.10-2.98)	0.0216	0.2592	1.74 (1.11-4.44)
ACE2 rs2285666	0.4708	>0.9999	0.85 (0.56-1.29)	0.9219	>0.9999	1.02 (0.69-1.49)
ABCG2 rs2231142	0.6666	>0.9999	1.12 (0.65-1.92)	0.8968	>0.9999	1.03 (0.62-1.70)
ATP2B4 rs1541252	0.2682	>0.9999	1.33 (0.82-2.19)	0.3083	>0.9999	1.25 (0.79-1.99)
SLC2A1 rs1385129	0.6072	>0.9999	0.88 (0.57-1.32)	0.7013	>0.9999	0.91 (0.62-1.35)
RAVER1 rs74956615	0.0928	>0.9999	1.97 (0.91-4.27)	0.0072	0.0864	2.80 (1.50-5.33)
MUC5B rs35705950	0.4900	>0.9999	0.79 (0.22-1.38)	0.0019	0.0228	2.61 (1.50-4.53)
UGT2A1.2A2 rs7688383	0.3408	>0.9999	1.18 (0.85-1.65)	0.1199	>0.9999	1.28 (0.94-2.74)

Supplementary table 6C: *Odds ratio analysis of patients with asthma bronchiale, compared to the European MAF in the 1000 Genome (phase3 release V3+) and the ALFA (Release Version: 20230706150541) databases. Statistical analysis was performed by Fisher test with Odds ratio analysis. In all cases the Bonferroni corrections were also performed.*

Gene polymorphism	Ref.: 1000Genomes (EUR)			Ref.: ALFA (EUR)		
	p value	p value (Bonferroni correction)	OR (95% CI)	p value	p value (Bonferroni correction)	OR (95% CI)
DPP4_DT rs118098838	0.4400	>0.9999	1.88 (0.24-11.23)	0.2381	>0.9999	3.78 (2.15-21.17)
DPP9 rs2109069	0.3594	>0.9999	0.70 (0.19-1.30)	0.453	>0.9999	0.75 (0.40-1.40)
OAS3 rs10735079	0.8826	>0.9999	1.04 (0.58-1.87)	>0.9999	>0.9999	1.03 (0.40-1.140)
IFNAR2 rs2236757	0.2715	>0.9999	1.42 (0.79-2.58)	0.2211	>0.9999	1.43 (0.82-2.50)
LZTFL1 rs73064425	0.0044	0.0528	2.96 (1.47-5.76)	0.004	0.0480	2.87 (1.51-5.39)
ACE2 rs2285666	>0.9999	>0.9999	0.95 (0.49-1.82)	0.7325	>0.9999	1.14 (0.60-2.18)
ABCG2 rs2231142	>0.9999	>0.9999	80 (0.30-2.11)	0.8176	>0.9999	0.73 (0.26-2.02)
ATP2B4 rs1541252	0.4864	>0.9999	1.33 (0.59-3.02)	0.5093	>0.9999	1.25 (0.57-2.78)
SLC2A1 rs1385129	0.7329	>0.9999	0.85 (0.21-1.72)	0.8656	>0.9999	0.88 (0.44-1.76)
RAVER1 rs74956615	0.6681	>0.9999	1.30 (0.30-4.85)	0.3018	>0.9999	1.86 (0.45-6.63)
MUC5B rs35705950	0.6462	>0.9999	0.68 (0.26-1.78)	0.1168	>0.9999	2.24 (0.86-5.99)
UGT2A1.2A2 rs7688383	0.7682	>0.9999	0.87 (0.48-1.58)	0.8854	>0.9999	0.94 (0.53-1.67)

Supplementary material 7:

Distribution of diabetes and asthma comorbidities among COVID-19 patients, grouped by COVID severity. Percentages shown in COVID severity groups.

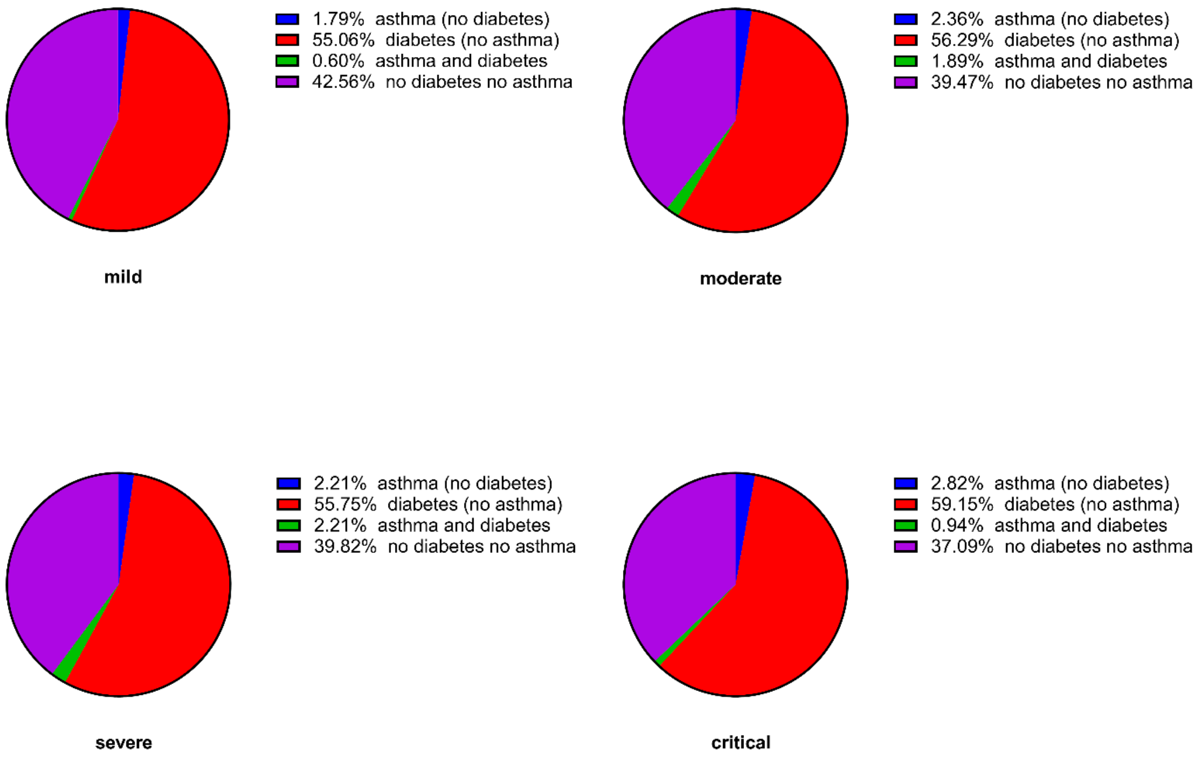

Supplement: S1 File — (PDF) [file pone.0316396.s001.pdf]
